# *rac*-(6*S*)-6-Hy­droxy-6-{2-[2-(propan-2-yl­idene)hydrazinyl­idene]prop­yl}indolo[2,1-*b*]quinazolin-12(6*H*)-one

**DOI:** 10.1107/S1600536811037408

**Published:** 2011-09-30

**Authors:** Matthew E. Rodstein, Paul D. Steffen, Bogdana Krivogorsky, Peter Grundt

**Affiliations:** a Department of Chemistry and Biochemistry, University of Minnesota Duluth, 1039 University Drive, Duluth, MN 55812, USA

## Abstract

The chiral title compound, C_21_H_20_N_4_O_2_, crystallizes as a racemic mixture. In the crystal, mol­ecules form centrosymmetric π-overlapping dimers [inter­planar distance = 3.338 (6) Å], which are further connected along the *a* axis forming centrosymmetric dimers *via* O—H⋯N hydrogen bonds. C—H⋯O inter­actions are also observed. The indolo[2,1-*b*]quinazoline group is somewhat bent, with a small dihedral angle of 6.3 (4)° between the plane of the quinazoline system and the plane of the benzene ring of the indole moiety. The C=N—N=C atoms of the azine group is oriented almost perpendicular [84.1 (2)°] to the mean plane of the quinazoline system.

## Related literature

The title compound is a derivative of the natural product tryptanthrin (indolo[2,1-*b*]quinazoline-6,12-dione). For reactions occurring at the 6-keto group of tryptanthrin with nucleophiles including CH-acidic compounds, see: Grandolini *et al.* (1997 ▶); Bergman & Tilstam (1985 ▶); Jao *et al.* (2008 ▶); Zou & Huang (1985 ▶). For related strutures, see: Brufani *et al.* (1971 ▶); Bergman *et al.* (1987 ▶); Jao *et al.* (2008 ▶); Grundt *et al.* (2010 ▶). For the Chebychev weighting scheme, see: Prince (1982 ▶); Watkin (1994 ▶).
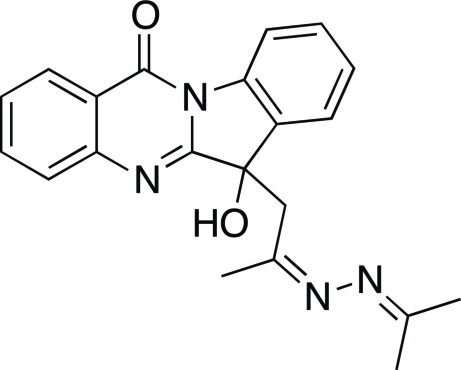


## Experimental

### 

#### Crystal data

C_21_H_20_N_4_O_2_
*M* *_r_* = 360.42
Monoclinic, 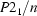

*a* = 8.6788 (17) Å
*b* = 15.117 (3) Å
*c* = 13.283 (3) Å
β = 99.58 (3)°
*V* = 1718.4 (6) Å^3^
*Z* = 4
Mo *K*α radiation
μ = 0.09 mm^−1^
*T* = 100 K
0.20 × 0.20 × 0.20 mm

#### Data collection

Rigaku R-AXIS RAPID II image-plate diffractometer
Absorption correction: multi-scan (*ABSCOR*; Higashi, 1995 ▶) *T*_min_ = 0.98, *T*_max_ = 0.98
11165 measured reflections
2914 independent reflections
1850 reflections with *I* > 2σ(*I*)
*R*_int_ = 0.096

#### Refinement

*R*[*F* ^2^ > 2σ(*F* ^2^)] = 0.062
*wR*(*F* ^2^) = 0.161
*S* = 1.01
2899 reflections
244 parameters
Only H-atom displacement parameters refined
Δρ_max_ = 0.59 e Å^−3^
Δρ_min_ = −0.48 e Å^−3^

### 

Data collection: *CrystalClear* (Rigaku Americas, 2009 ▶); cell refinement: *HKL-2000* (Otwinowski & Minor, 1997 ▶); data reduction: *CrystalClear*; program(s) used to solve structure: *CrystalStructure* (Rigaku Americas, 2009 ▶) and *SIR2004* (Burla *et al.*, 2005 ▶); program(s) used to refine structure: *CRYSTALS* (Betteridge *et al.*, 2003 ▶); molecular graphics: *CAMERON* (Watkin *et al.*, 1996 ▶); software used to prepare material for publication: *CRYSTALS*.

## Supplementary Material

Crystal structure: contains datablock(s) global, I. DOI: 10.1107/S1600536811037408/ld2027sup1.cif

Structure factors: contains datablock(s) I. DOI: 10.1107/S1600536811037408/ld2027Isup2.hkl

Supplementary material file. DOI: 10.1107/S1600536811037408/ld2027Isup3.cml

Additional supplementary materials:  crystallographic information; 3D view; checkCIF report

## Figures and Tables

**Table 1:** Hydrogen-bond geometry (Å, °)

*D*—H⋯*A*	*D*—H	H⋯*A*	*D*⋯*A*	*D*—H⋯*A*
C20—H10⋯O18^i^	0.98	2.57	3.381 (6)	140 (1)
O19—H9⋯N5^ii^	0.83	2.08	2.872 (6)	160 (1)

Symmetry codes: (i) 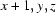; (ii) 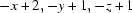.

**Fig. 1.**
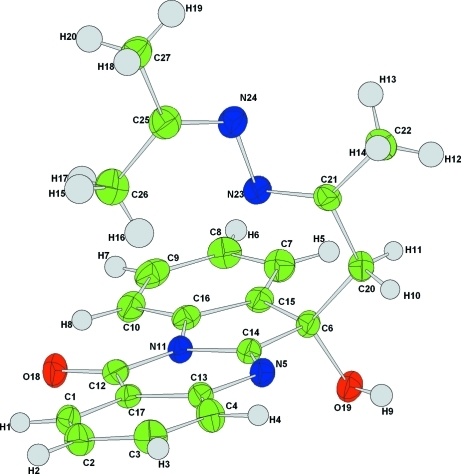
The title compound with displacement ellipsoids drawn at the 50% probability level. H atoms are shown as spheres of arbitary radius.

**Fig. 2.**
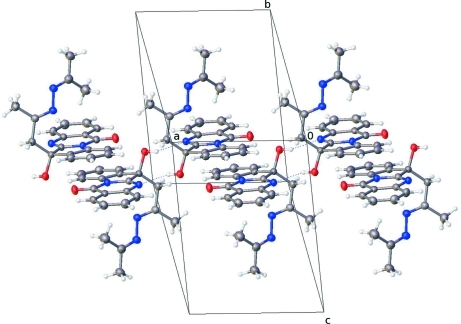
Packing diagram for the title compound showing intermolecular O—H···N bonds.

## supplementary crystallographic information

### Comment

The 6-keto group of the natural product tryptanthrin (indolo[2,1-*b*]quinazoline-6,12-dione) has been shown to react with numerous nucleophiles including CH-acidic compounds (Grandolini *et al.*, 1997, Bergman & Tilstam, 1985, Jao *et al.*, 2008, Zou & Huang, 1985). The title compound was obtained by reacting tryptanthrin with hydrazine in acetone as a solvent.

In the structure of the title compound, the azine moiety was determined to possess *E*-configuration in respect to the C21=N23 double bond with a *trans*-orientation around the N23—N24 bond (dihedral angle 158.6 (4)°). The CN double bonds of the azine moiety were found to be slighly shorter than the corresponding conjugated CN bonds in the quinazoline system. The C=O bond clearly has double bond character and was observed to be 1.226 (4) Å in length.

### Experimental

1.0 mL (20 mmol) hydrazine hydrate was added dropwise to a suspension of 0.25 g (1.0 mmol) tryptanthrin in 10 mL of acetone and the reaction mixture was heated to reflux for 30 min. Upon cooling the title compound crystallized from the reaction mixture. The precipitate was collected and washed with a small amount of acetone to give 0.26 g (72%) of the title compound I. Crystals suitable for X-ray analysis were grown by slow diffusion of hexane into a solution of the title compound in ethylacetate/chloroform 1:1. The crystal was diffracted in the cold stream of an X-Stream2000 Liquid nitrogen generator with an open-flow nitrogen cryostat with a nominal stability of 0.1°K.

### Refinement

Only hkl indices better than 0.85 Å resolution were integrated. The H atoms - except O-H - were all located in a difference map, but were repositioned geometrically. The positions of Me groups were optimized rotationally using default algorithm implemented in the *CRYSTALS* software. The H atoms were initially refined with soft restraints on the bond lengths and angles to regularize their geometry (C—H in the range 0.93–0.94 Å, O—H in the range 0.82–0.84 Å, O) and U_iso(_H) (in the range 1.2–1.5 times *U*_eq_ of the parent atom), after which the positions were refined with riding constraints.

### Crystal data

**Table:**

C_21_H_20_N_4_O_2_	*F*(000) = 760
*M**_r_* = 360.42	*D*_x_ = 1.393 Mg m^−^^3^
Monoclinic, *P*2_1_/*n*	Mo *K*α radiation, λ = 0.71073 Å
Hall symbol: -P 2yn	Cell parameters from 1820 reflections
*a* = 8.6788 (17) Å	θ = 25–2°
*b* = 15.117 (3) Å	µ = 0.09 mm^−^^1^
*c* = 13.283 (3) Å	*T* = 100 K
β = 99.58 (3)°	Block, colourless
*V* = 1718.4 (6) Å^3^	0.20 × 0.20 × 0.20 mm
*Z* = 4	

### Data collection

**Table:**

Rigaku R-AXIS RAPID II image-plate diffractometer	2914 independent reflections
Radiation source: Mo Sealed tube tube	1850 reflections with *I* > 2σ(*I*)
graphite	*R*_int_ = 0.096
Detector resolution: 10 pixels mm^-1^	θ_max_ = 24.7°, θ_min_ = 3.1°
ω/2θ scans	*h* = −10→9
Absorption correction: multi-scan (*ABSCOR*; Higashi, 1995)	*k* = −17→17
*T*_min_ = 0.98, *T*_max_ = 0.98	*l* = −14→15
11165 measured reflections	

### Refinement

**Table:**

Refinement on *F*^2^	Primary atom site location: structure-invariant direct methods
Least-squares matrix: full	Secondary atom site location: difference Fourier map
*R*[*F*^2^ > 2σ(*F*^2^)] = 0.062	Hydrogen site location: inferred from neighbouring sites
*wR*(*F*^2^) = 0.161	Only H-atom displacement parameters refined
*S* = 1.01	Method, part 1, Chebychev polynomial,(Watkin, 1994; *P*rince, 1982) [*w*eight] = 1.0/[A_0_*T_0_(x) + A_1_*T_1_(x) ··· + A_n-1_]*T_n-1_(x)] where A_i_ are the Chebychev coefficients listed belo*w* and x = *F* /*F*max Method = Robust Weighting (*P*rince, 1982) W = [*w*eight] * [1-(delta*F*/6*sigma*F*)^2^]^2^ A_i_ are: 5.56 6.74 1.72
2899 reflections	(Δ/σ)_max_ = 0.0001471
244 parameters	Δρ_max_ = 0.59 e Å^−^^3^
0 restraints	Δρ_min_ = −0.48 e Å^−^^3^
13 constraints	

### Special details

**Table:**

Experimental. ^1^H NMR (DMSO-d^6^, 500 MHz): δ 0.82 (s, 3H), 1.63 (s, 6H), 3.38 (d, J 16.6, 1H), 3.44 (d, J 16.5, 1H), 6.88 (s, 1H), 7.35 (t, J 7.3, 1H), 7.46 (t, J 7.7, 1H), 7.59 (t, J 7.2, 1H), 7.62 (d, J 6.7, 1H), 7.78 (d, J 8.0, 1H), 7.87 (t, J 8.3, 1H), 8.28 (d, J 7.8, 1H), 8.39 (d, J 7.7, 1H). ^13^C NMR (DMSO-d^6^, 125 MHz): δ 16.5, 17.3, 24.3, 45.5, 75.3, 115.9, 121.2, 123.4, 126.2, 126.3, 127.0, 127.3, 129.2, 134.4, 134.6, 139.2, 147.2, 157.6, 158.9, 159.0, 161.2.
Refinement. Crystals for Windows program eliminates all reflections with [sinθ/λ]^2^ < 0.01 in order to eliminate reflections that may be poorly measured in the vicinity of the beam stop. Such filter eliminated 15 reflections, which resulted in difference between 2914 measured unique reflections and 2899 reflections used for refinement.

### Fractional atomic coordinates and isotropic or equivalent isotropic displacement parameters (Å^2^)

**Table:**

	*x*	*y*	*z*	*U*_iso_*/*U*_eq_	
O19	0.8991 (3)	0.37375 (18)	0.4708 (2)	0.0293	
C6	0.8330 (4)	0.4137 (3)	0.3753 (3)	0.0247	
C14	0.7388 (4)	0.4957 (2)	0.3956 (3)	0.0236	
N11	0.5809 (3)	0.4758 (2)	0.3731 (2)	0.0246	
C16	0.5590 (4)	0.3890 (2)	0.3310 (3)	0.0244	
C15	0.7034 (4)	0.3519 (3)	0.3277 (3)	0.0252	
C7	0.7135 (5)	0.2688 (3)	0.2869 (3)	0.0333	
C8	0.5753 (5)	0.2223 (3)	0.2529 (3)	0.0340	
C9	0.4329 (5)	0.2595 (3)	0.2605 (3)	0.0344	
C10	0.4209 (5)	0.3440 (3)	0.2997 (3)	0.0310	
C12	0.4659 (4)	0.5369 (3)	0.3857 (3)	0.0259	
O18	0.3266 (3)	0.51841 (19)	0.3656 (2)	0.0338	
C17	0.5287 (4)	0.6229 (3)	0.4227 (3)	0.0252	
C13	0.6913 (4)	0.6366 (3)	0.4431 (3)	0.0258	
N5	0.7978 (3)	0.5697 (2)	0.4294 (2)	0.0260	
C4	0.7479 (5)	0.7207 (3)	0.4739 (3)	0.0312	
C3	0.6455 (5)	0.7880 (3)	0.4861 (3)	0.0356	
C2	0.4848 (5)	0.7732 (3)	0.4689 (3)	0.0348	
C1	0.4268 (4)	0.6917 (3)	0.4381 (3)	0.0294	
C20	0.9621 (4)	0.4328 (3)	0.3137 (3)	0.0272	
C21	0.9162 (4)	0.4779 (2)	0.2122 (3)	0.0258	
N23	0.7732 (4)	0.4958 (2)	0.1820 (2)	0.0301	
N24	0.7448 (4)	0.5367 (2)	0.0842 (3)	0.0341	
C25	0.6178 (5)	0.5803 (3)	0.0657 (3)	0.0370	
C27	0.5820 (5)	0.6268 (3)	−0.0346 (3)	0.0413	
C26	0.5009 (6)	0.5914 (4)	0.1369 (4)	0.0487	
C22	1.0477 (5)	0.4992 (3)	0.1570 (3)	0.0363	
H5	0.8117	0.2442	0.2822	0.0396*	
H6	0.5785	0.1667	0.2260	0.0411*	
H7	0.3403	0.2271	0.2407	0.0409*	
H8	0.3245	0.3690	0.3040	0.0364*	
H4	0.8560	0.7312	0.4877	0.0372*	
H3	0.6855	0.8437	0.5051	0.0417*	
H2	0.4150	0.8196	0.4786	0.0419*	
H1	0.3181	0.6816	0.4280	0.0349*	
H10	1.0397	0.4707	0.3542	0.0334*	
H11	1.0097	0.3759	0.3019	0.0321*	
H20	0.4768	0.6120	−0.0675	0.0614*	
H18	0.5915	0.6909	−0.0245	0.0623*	
H19	0.6553	0.6078	−0.0779	0.0617*	
H15	0.4565	0.6506	0.1315	0.0720*	
H16	0.5516	0.5824	0.2069	0.0725*	
H17	0.4179	0.5475	0.1194	0.0723*	
H13	1.0232	0.4842	0.0867	0.0554*	
H14	1.0673	0.5604	0.1619	0.0561*	
H12	1.1415	0.4695	0.1848	0.0554*	
H9	0.9793	0.4019	0.4925	0.0440*	

### Atomic displacement parameters (Å^2^)

**Table:**

	*U*^11^	*U*^22^	*U*^33^	*U*^12^	*U*^13^	*U*^23^
O19	0.0221 (13)	0.0337 (15)	0.0307 (15)	−0.0019 (12)	0.0004 (11)	0.0048 (12)
C6	0.0199 (18)	0.030 (2)	0.0239 (19)	0.0019 (16)	0.0029 (15)	0.0069 (16)
C14	0.0199 (18)	0.029 (2)	0.0214 (18)	−0.0007 (15)	0.0025 (14)	0.0016 (16)
N11	0.0198 (15)	0.0290 (17)	0.0247 (16)	−0.0018 (13)	0.0028 (12)	0.0013 (14)
C16	0.0276 (19)	0.025 (2)	0.0200 (18)	−0.0013 (16)	0.0023 (15)	0.0037 (15)
C15	0.0254 (19)	0.028 (2)	0.0224 (19)	−0.0033 (16)	0.0051 (15)	0.0065 (16)
C7	0.033 (2)	0.031 (2)	0.036 (2)	0.0001 (18)	0.0052 (17)	0.0031 (18)
C8	0.039 (2)	0.029 (2)	0.034 (2)	−0.0014 (18)	0.0058 (18)	−0.0013 (18)
C9	0.037 (2)	0.036 (2)	0.027 (2)	−0.0116 (19)	−0.0015 (17)	0.0008 (18)
C10	0.026 (2)	0.036 (2)	0.030 (2)	−0.0031 (17)	0.0006 (16)	0.0058 (18)
C12	0.023 (2)	0.033 (2)	0.0212 (19)	−0.0011 (16)	0.0028 (15)	0.0003 (16)
O18	0.0193 (14)	0.0423 (17)	0.0384 (16)	−0.0002 (12)	0.0011 (12)	−0.0022 (13)
C17	0.0224 (19)	0.034 (2)	0.0189 (18)	0.0002 (16)	0.0035 (14)	0.0042 (16)
C13	0.0230 (19)	0.030 (2)	0.0244 (19)	0.0033 (16)	0.0049 (15)	0.0058 (16)
N5	0.0210 (16)	0.0290 (18)	0.0278 (17)	−0.0004 (14)	0.0033 (13)	0.0038 (14)
C4	0.026 (2)	0.029 (2)	0.039 (2)	−0.0041 (17)	0.0069 (17)	0.0003 (18)
C3	0.038 (2)	0.028 (2)	0.040 (2)	−0.0015 (18)	0.0060 (19)	−0.0028 (18)
C2	0.034 (2)	0.035 (2)	0.036 (2)	0.0072 (19)	0.0065 (18)	0.0013 (19)
C1	0.0231 (19)	0.035 (2)	0.030 (2)	0.0068 (17)	0.0040 (16)	0.0073 (17)
C20	0.0226 (19)	0.028 (2)	0.031 (2)	0.0020 (16)	0.0040 (16)	0.0020 (16)
C21	0.028 (2)	0.025 (2)	0.0245 (19)	0.0039 (16)	0.0060 (16)	−0.0017 (16)
N23	0.0278 (18)	0.0352 (19)	0.0271 (17)	0.0024 (15)	0.0042 (14)	0.0050 (15)
N24	0.0301 (18)	0.039 (2)	0.0329 (19)	0.0024 (16)	0.0034 (15)	0.0086 (16)
C25	0.034 (2)	0.040 (2)	0.037 (2)	−0.004 (2)	0.0052 (18)	0.003 (2)
C27	0.037 (2)	0.048 (3)	0.037 (2)	0.004 (2)	0.0009 (19)	0.008 (2)
C26	0.044 (3)	0.058 (3)	0.044 (3)	0.011 (2)	0.007 (2)	0.004 (2)
C22	0.032 (2)	0.043 (3)	0.035 (2)	0.0011 (19)	0.0101 (18)	−0.001 (2)

### Geometric parameters (Å, °)

**Table:**

O19—C6	1.436 (4)	C4—C3	1.379 (6)
O19—H9	0.826	C4—H4	0.939
C6—C14	1.532 (5)	C3—C2	1.393 (6)
C6—C15	1.518 (5)	C3—H3	0.929
C6—C20	1.520 (5)	C2—C1	1.367 (6)
C14—N11	1.386 (5)	C2—H2	0.950
C14—N5	1.281 (5)	C1—H1	0.942
N11—C16	1.426 (5)	C20—C21	1.505 (5)
N11—C12	1.391 (5)	C20—H10	0.975
C16—C15	1.380 (5)	C20—H11	0.979
C16—C10	1.381 (5)	C21—N23	1.268 (5)
C15—C7	1.377 (6)	C21—C22	1.491 (5)
C7—C8	1.399 (6)	N23—N24	1.423 (4)
C7—H5	0.941	N24—C25	1.272 (5)
C8—C9	1.377 (6)	C25—C27	1.493 (6)
C8—H6	0.916	C25—C26	1.507 (6)
C9—C10	1.390 (6)	C27—H20	0.971
C9—H7	0.941	C27—H18	0.979
C10—H8	0.928	C27—H19	0.969
C12—O18	1.226 (4)	C26—H15	0.973
C12—C17	1.463 (5)	C26—H16	0.970
C17—C13	1.408 (5)	C26—H17	0.979
C17—C1	1.402 (5)	C22—H13	0.950
C13—N5	1.401 (5)	C22—H14	0.942
C13—C4	1.399 (5)	C22—H12	0.948
			
C6—O19—H9	106.3	C3—C4—H4	119.7
O19—C6—C14	109.3 (3)	C4—C3—C2	120.6 (4)
O19—C6—C15	105.5 (3)	C4—C3—H3	118.8
C14—C6—C15	101.0 (3)	C2—C3—H3	120.6
O19—C6—C20	109.4 (3)	C3—C2—C1	120.2 (4)
C14—C6—C20	113.8 (3)	C3—C2—H2	120.2
C15—C6—C20	117.0 (3)	C1—C2—H2	119.6
C6—C14—N11	108.9 (3)	C17—C1—C2	120.1 (4)
C6—C14—N5	125.1 (3)	C17—C1—H1	120.0
N11—C14—N5	126.0 (3)	C2—C1—H1	119.9
C14—N11—C16	110.3 (3)	C6—C20—C21	117.3 (3)
C14—N11—C12	122.3 (3)	C6—C20—H10	108.6
C16—N11—C12	127.4 (3)	C21—C20—H10	106.4
N11—C16—C15	108.9 (3)	C6—C20—H11	106.8
N11—C16—C10	128.6 (4)	C21—C20—H11	108.1
C15—C16—C10	122.5 (4)	H10—C20—H11	109.6
C6—C15—C16	110.5 (3)	C20—C21—N23	118.6 (3)
C6—C15—C7	129.4 (4)	C20—C21—C22	115.4 (3)
C16—C15—C7	120.1 (4)	N23—C21—C22	126.0 (4)
C15—C7—C8	118.5 (4)	C21—N23—N24	113.2 (3)
C15—C7—H5	120.3	N23—N24—C25	114.4 (3)
C8—C7—H5	121.2	N24—C25—C27	117.4 (4)
C7—C8—C9	120.2 (4)	N24—C25—C26	126.1 (4)
C7—C8—H6	120.4	C27—C25—C26	116.5 (4)
C9—C8—H6	119.3	C25—C27—H20	109.5
C8—C9—C10	121.8 (4)	C25—C27—H18	110.1
C8—C9—H7	120.1	H20—C27—H18	109.9
C10—C9—H7	118.0	C25—C27—H19	109.3
C9—C10—C16	116.7 (4)	H20—C27—H19	109.0
C9—C10—H8	121.4	H18—C27—H19	109.0
C16—C10—H8	121.9	C25—C26—H15	110.9
N11—C12—O18	121.6 (4)	C25—C26—H16	109.9
N11—C12—C17	113.4 (3)	H15—C26—H16	108.1
O18—C12—C17	125.0 (4)	C25—C26—H17	108.7
C12—C17—C13	120.0 (3)	H15—C26—H17	109.8
C12—C17—C1	120.0 (3)	H16—C26—H17	109.4
C13—C17—C1	120.0 (4)	C21—C22—H13	111.6
C17—C13—N5	122.1 (4)	C21—C22—H14	108.7
C17—C13—C4	118.8 (3)	H13—C22—H14	108.1
N5—C13—C4	119.1 (3)	C21—C22—H12	112.6
C13—N5—C14	116.3 (3)	H13—C22—H12	107.9
C13—C4—C3	120.2 (4)	H14—C22—H12	107.8
C13—C4—H4	120.1		

### Hydrogen-bond geometry (Å, °)

**Table:**

*D*—H···*A*	*D*—H	H···*A*	*D*···*A*	*D*—H···*A*
C20—H10···O18^i^	0.98	2.57	3.381 (6)	140.(1)
O19—H9···N5^ii^	0.83	2.08	2.872 (6)	160.(1)

Symmetry codes: (i) *x*+1, *y*, *z*; (ii) −*x*+2, −*y*+1, −*z*+1.
